# Training in psychiatry throughout Europe

**DOI:** 10.1007/s00406-016-0679-4

**Published:** 2016-02-15

**Authors:** Andrew Brittlebank, Marc Hermans, Dinesh Bhugra, Mariana Pinto da Costa, Martina Rojnic-Kuzman, Andrea Fiorillo, Tamas Kurimay, Cecile Hanon, Danuta Wasserman, Rutger Jan van der Gaag

**Affiliations:** Cumbria Partnership NHS Foundation Trust, Cumbria, UK; Royal College of Psychiatrists, London, UK; Mechelen, Sint-Niklaas, Belgium; Institute of Psychiatry, Psychology & Neuroscience, King’s College London, London, UK; Hospital de Magalhães Lemos, University of Porto, Porto, Portugal; Zagreb School of Medicine and Zagreb University Hospital Centre, Zagreb, Croatia; Department of Psychiatry, University of Naples SUN, Naples, Italy; Institute of Behaviour Sciences, Semmelweis University, Budapest, Hungary; Centre Ressource Régional de Psychiatrie du sujet âgé - Hôpital Corentin Celton Hôpitaux, Universitaires Paris-Ouest, Nanterre, France; National Centre for Prevention of Suicide and Mental Ill-health (NASP), Stockholm, Sweden; WHO Collaborating Centre for Research, Methods Development and Training in Suicide Prevention, Stockholm, Sweden; Department of Psychiatry, Radboud University Medical Centre, Nijmegen, The Netherlands

**Keywords:** Education, Training, Psychiatry, Psychotherapy, Crosscultural, European standardisation

## Abstract

Psychiatry is the largest medical specialty in Europe. Despite efforts to bring harmonisation, training in psychiatry in Europe continues to be very diverse. The Union Européenne des Médecins Spécialistes (UEMS) has issued as from 2000 a charter of requirements for the training in psychiatry with an additional European Framework for Competencies in Psychiatry in 2009. Yet these have not been implemented throughout Europe. In this paper, the diversity in training throughout Europe is approached from different angles: the cultural differences between countries with regards to how mental health care is considered and founded on, the cultural differences between people throughout Europe in all states. The position of psychotherapy is emphasised. What once was the cornerstone of psychiatry as medical specialty seems to have become a neglected area. Seeing the patient with mental health problems within his cultural context is important, but considering him within his family context. The purpose of any training is enabling the trainee to gain the knowledge and acquire the competencies necessary to become a well-equipped professional is the subject of the last paragraph in which trainees consider their position and early career psychiatrists look back to see whether what they were trained in matches with what they need in the working situation. Common standard for training and certification are a necessity within Europe, for the benefit of the profession of psychiatrist but also for patient safety. UEMS is advised to join forces with the Council of National Psychiatric Associations (NPAs) within the EPA and trainings and early career psychiatrist, to discuss with the users what standards should be implemented in all European countries and how a European board examination could ensure professional quality of psychiatrists throughout the continent.

## Introduction

### Scope and purpose of this guidance paper

Psychiatry is the largest medical specialty in Europe. Training to become a psychiatrist begins after the doctor has been awarded a licence to practise medicine and continues until the doctor is recognised as a specialist. Despite efforts to bring harmonisation, training in psychiatry in Europe continues to be very diverse. The diversity is seen in several respects: throughout Europe, there is great variability in the position of the trainee, the duration and content of training and how to gain and maintain recognition as a specialist.

The reason why harmonisation of training is an important issue within the European Union (EU) is embedded in the founding principles of the EU in the free movement of goods, capital, services and labour. Consequently, there is a need for mutual recognition of professional qualifications. This necessarily imposes a process of standardisation in training programmes, competency and quality assessment for the safety of patients and the security of professionals while respecting existing cultural diversity.

In addition to the issue of harmonisation is the pressing problem in many European countries of the poor recruitment of medical graduates into psychiatry leading to a shortfall in the number needed to maintain an adequate medical labour force in mental health services.

Mayer et al. [1] conducted an extensive literature search to find primary evidence that measured the diversity of training in Europe. As a result of this search, the authors found only six original papers that met their search criteria, of which four described survey data. In addition, the authors identified six published postgraduate curricula that they were able to describe. From this evidence, the authors concluded that there is considerable difference in training experience throughout Europe that will present barriers to the goal of harmonisation. They recommended that there should be greater publication and standardisation of curricula and suggested that there should be an agreed assessment process that leads to the qualification of “Fellow of the European Board of Psychiatry”.

The dearth of evidence concerning trainee psychiatrists’ educational experience in Europe is disappointing, but hardly surprising given what is known about scholarship in medical education which indicates that published research rarely addresses issues of effectiveness [2]. Furthermore, as Jan Illing [3] pointed out in medical education, acceptable forms of evidence are garnered from sources that may be different from those that are found in the traditional biomedical literature. Acceptable methodologies may consist of the qualitative, including the narrative. The narrative knowledge of experts by experience and experts by practice, especially when verified through triangulation, may thus be legitimate forms of evidence.

Mayer et al’s review addresses the question of harmonisation, but does not consider the question of what the harmonisation should focus upon. There is little point in every European nation offering the same experience to psychiatrists in training, if that experience fails to produce doctors who can function effectively to provide services that mentally ill people in Europe need.

This guidance paper seeks to build on the work of Mayer et al. [1] by exploring the issue of a common European standard for training in psychiatry from different perspectives:

- A description of the evidence used to develop European standards and the extent to which they are implemented
- The issue of trans-cultural competence in psychiatry and how training psychiatrists to be more culturally competent will support the transferability of psychiatric expertise between different cultures
- A systemic approach that supports the psychiatrist to see illness from a family and carer orientation.
- The position of formal psychotherapy training within training programmes

Finally, we will use a conceptual framework taken from the field of organisational science to explore why efforts to harmonise training have failed and from this to make recommendations that may have more success than those that have been used so far. We will use this to argue for a greater inclusivity in the discussions around harmonising and improving training.

This guidance paper is meant to address those mostly involved: trainees and trainers. More indirectly we also intend to reach national and international professional bodies responsible for training development and evaluation.

Following Illing’s [3] injunction to use multiple sources of evidence, this guidance will consider several types of evidence, including that derived from experts by experience and from expert practitioners in the field.

### Training in psychiatry across Europe?

The Treaty of Rome, signed in 1957, brought into being the European Economic Community (EEC). It created a common market allowing the free movement of persons, services, goods and capital within its member states. The mutual recognition of professional qualifications was an essential prerequisite of the free movement of professionals. From 1975, member states of the EEC were required to mutually recognise basic and specialist medical qualifications. This requirement was consolidated in Directive 93/16/EEC [4], enacted on 5th April 1993. The Directive requires member states to recognise basic medical qualifications awarded in other member states, and it stipulated that basic medical training should be of at least 6-year duration.

The Union Européenne de Médecins Spécialistes (UEMS, unofficially *European Union of Medical Specialists*) was founded in 1958 by professional organisations of medical specialists within the EEC. The UEMS in supporting the concept of free movement of medical specialists in the EEC set out to secure the quality of training, continuous medical education and professional development, and quality of practice for all specialties. It quickly became involved in quality-improving initiatives. This is culminated in the publication of the Charter on Training of Medical Specialists in the European Community in October 1993 [5]. This Charter sets out requirements for the training that was thought to be necessary to prepare doctors for the appropriate level of specialist practice in any member state. The requirements were set out in six parts. The first five describe general requirements for all programmes of specialist training. The so-called Chapter Six, written by the UEMS Board of the specialty concerned, describes the quality standards needed for training in a particular specialty.

The UEMS Board of Psychiatry was established in 1992. The Board of Psychiatry published the psychiatry specialist training chapter of the Charter in April 2000 [6]. This chapter sets requirements for the duration, organisation, content and quality control of psychiatry specialist training. Summarising, the chapter stipulates that training should cover a minimum of 5 years; during their training, trainees should rotate between clinical services and treat different psychiatric disorders; trainees should have experience in general adult psychiatry, old age psychiatry, substance misuse psychiatry, developmental psychiatry as well as supervised experience of psychotherapy; there should be established internal systems of quality assurance within training institutions.

Despite the effort put into the production of UEMS requirements, their impact was limited by their advisory nature, the lack of resources available to the Board of Psychiatry and the diversity of mental health provision in Europe [7]. Surveys of training institutions and of trainees conducted in the years following the publication of the Chapter Six of the UEMS Charter showed that only the basic requirements of the EEC Directive were consistently met and that there was a significant variation in the content and structure of training delivered around Europe as well as in methods of assessing training outcomes and of quality assuring training [8–10]. More disturbingly, differences in understanding of the meaning and in the delivery of mental health services may conceal the true magnitude of this variation [11].

In order to address these issues of inconsistency, the next step of the UEMS Board of Psychiatry was to produce curriculum guidelines. This was to be based on a European consensus statement on the core competencies of a psychiatrist [12]. The European Framework for Competencies in Psychiatry (EFCP) [13] was published in 2009 following an iterative development process that engaged key stakeholders including psychiatric educators, national psychiatric associations, psychiatrists in training and users and carers of people who use mental health services. The level of engagement of experts by experience (trainees, users and carers) in the development of the EFCP strengthens the validity of the content of the EFCP as a statement of what the European Specialist Psychiatrist should be able to do by the end of training.

The EFCP uses the seven physician roles^1^ from the CanMEDS [14] as “metacompetencies”, headers for 26 intended learning outcomes, termed “key competencies”, along with their supporting competencies. The key competencies of the EFCP are displayed in Table 1.

**Table 1:** Seven CanMEDS physician roles and their associated key competencies from the European framework for competencies in psychiatry

Physician role	Key competencies
1. Psychiatric expert/clinical decision-maker is able to	1.1 conceptualise, understand and apply the diagnostic skills to investigate, elicit, describe and define psychopathological and other clinical findings 1.2 apply therapeutic skills to effectively and ethically manage the spectrum of patient care problems diagnosed 1.3 apply psychiatric expertise in situations other than in direct patient care 1.4 recognise personal limits of expertise 1.5 consult effectively
2. Communicator is able to	2.1 establish a therapeutic relationship with patients 2.2 elicit and synthesise relevant information from the patient, their carers and other relevant sources 2.3 discuss appropriate information with the patient, their carers and health professionals that facilitate optimal care. This implies the ability to inform and counsel a patient in a sensitive and respectful manner while fostering understanding the patient’s active participation in decisions about their care.
3. Collaborator is able to	3.1 effectively consult with other physicians and healthcare professionals 3.2 contribute effectively to other interdisciplinary team activities 3.3 participate actively in shared decision making with patients and carers 3.4 collaborate effectively with patient and carer organisations
4. Manager is able to	4.1 allocate limited healthcare resources 4.2 manage personal resources 4.3 work in a healthcare organisation 4.4 use information technology to optimise patient care, continued self-learning and other activities
5. Health advocate is able to	5.1 identify the determinants of mental disorder as well as the factors that may contribute to positive mental health so as to be able to prevent disorder and promote mental health 5.2 identify and address issues and circumstances when advocacy on behalf of patients, professions, or society is necessary
6. Scholar is able to	6.1 develop, implement and document a personal continuing education strategy 6.2 apply the principles of critical appraisal to sources of medical information 6.3 facilitate learning in patients, students, trainees and health professionals 6.4 facilitate the learning of colleagues, trainees and students through the appropriate use of assessment, appraisal and feedback 6.5 contribute to research and to the development of new knowledge
7. Professional is able to	7.1 deliver the highest quality of professional care 7.2 relate to co-workers in a professional manner 7.3 practise medicine in an ethically responsible manner that respects medical, legal and professional obligations

The EFCP also includes a grid of methods suggested to assess each supporting competency. These include a range of knowledge tests, clinical examinations and in-training assessments of performance. Table 2 illustrates how the key competencies, supporting competencies and suggested assessment methods fit together in the curriculum area concerned with psychotherapy.

**Table 2:** Key competency in psychotherapy with its supporting competencies and their recommended assessment methods under the domains of knowledge, competence and performance, taken from the European framework for competencies in psychiatry

Supporting competencies	Knowledge Knowledge tests	Competence Clinical examinations	Performance In-training assessment
1.2.2.1 understand the theories that underpin standard accepted models of individual, group and family psychotherapies available for treatment of mental disorders	Oral examinations Written examinations		
1.2.2.2 practise psychotherapy safely and effectively on the basis of values and the best evidence available			Document-based discussions Direct observations of procedures

Key competency 1.2 the psychiatrist is able to apply therapeutic and communication skills to effectively and empathically manage the spectrum of patient care problems as well as those of their carers

There are no formal data describing the impact of the EFCP. There is anecdotal evidence that some national psychiatry training programmes have been constructed to deliver the outcomes of the EFCP. The overall indications are that the EFCP has had the same inconsistent impact as the UEMS Charter on Training.

The variation in different aspects of training is a concern in itself, but the greater concern is how this may be manifested as variation in the quality of psychiatric practice [11].

Psychiatry training is embedded in the mental health services that are available in a particular society and they in turn are embedded in the national and regional cultures of the society. Due to sensitivity to what they called “national conditions”, the authors of the EFCP deliberately avoided producing a curriculum based on the competencies in the framework. They leave it to local agencies to specify such details. Because a curriculum should describe methods of learning and progression within a particular training programme, it might be very much influenced by these national conditions.

There is large variation across Europe in the delivery of mental health services. According to the WHO in 2014 [15], the combined number of psychiatric beds in Europe per 100.000 population ranges from 185 in Malta to 8 in Italy. Admission rates to inpatient units per 100.000 inhabitants vary from 1.301 in Romania and 1.240 in Germany to 87 in Albania. The annual number of outpatient attendances per 100.000 inhabitants varies from 28.2 in Slovakia and 26.1 in Finland to 1.08 in Albania and 1.07 in the UK.

Similar variability is seen in workforce composition. The number of psychiatrists per 100.000 inhabitants varies from 30 per 100.000 in Switzerland and 26 in Finland to 3 in Albania and 1 in Turkey.

The number of nurses working in mental health care shows similar variations, from 163 per 100,000 population in Finland to 3 in Greece 7.

The variability in the composition of the mental health workforce will presumably be reflected in differences in the work that is taken on by different professions across Europe and this in turn will influence the content of postgraduate training.

### Cultural differences in training in psychiatry throughout Europe

Europe is a continent with a multitude of cultures and sub-cultures, which in the past decades has experienced a large influx of even more cultures from around the globe. These cultural differences will have as much an impact as the composition of the mental health workforce on how medicine and psychiatry are practised in different countries. They also underline the importance of training in cultural psychiatry. Formally, there is free movement of professionals across the European Union. But countries can ask for additional requirements as sufficient language skills in the hosting country and additional training if the requirements for the specialty are very different to those in the hosting country.

Harmonising of training in psychiatry in Europe should therefore also take into account the following aspects:

1. Differences in concepts regarding mental health and service delivery and the position of psychiatrists within the field of mental health.
2. Differences in board certification and the requirements for certification and licensing
3. Cultural differences in patients and their families in interaction with the culture of the medical staff

### Differences in concepts

When the culture of psychiatric practice around Europe is examined, some differences begin to emerge. In some countries, there is a tradition of associating psychiatry with psychoanalysis. In others, the impact of psychoanalysis on psychiatry has almost disappeared. This leads to tensions between different European and national psychiatric associations which represent the different traditions of neurobiology and psychoanalysis. This can lead to political and governing bodies exerting influence on the curricula and the registration/board examinations.

Central and Eastern European countries have a heritage of many decades of political regimes in which psychiatry was associated with “discretely eliminating from society” politically critical individuals. At the same time, psychiatry was the ill-favoured branch of health care with a tradition of institutionalisation with very low budgets and archaic circumstances. Though the conditions have dramatically changed, the issue of stigma and discrimination of individuals with mental health problems is still strongly at stake.

Nowadays, the north-western part of Europe is characterised by a strongly empirically driven psychiatry, a research-driven medical specialty that is gaining influence at the universities, in society and political spheres.

Thus, one is in fact faced with a continent with very different “psychiatry’s”, what makes setting Europe-wide standards quite a challenge. Although it would not fit to impose one single model within Europe, a consensus on values common to all could form the basis and could, within accepted limits, be extended with local values and habits. But those local cultural differences will need to be explored in detail, in order to know how the certification should be adapted when a registered psychiatrist moves from one part of the continent to another.

## Cultural differences

Every individual carries with them their cultural values, norms and mores. It is the culture in which we are born which moulds our world view and the way we think. Similarly, cultures that we live in influence the way we function, think, express ourselves and what idioms of distress we use in expressing our feelings of not being well. Society and cultures decide on what health care is available and how it is delivered. Doctors and patients both have cultural values and different ways of looking at what symptoms are being presented.

### Model of culture and individual

The EFCP references cultural competencies under the metacompetencies of the physician as psychiatric expert and as communicator. These focus on the importance of the psychiatrist in understanding their patient’s cultural background as well as the cultural context of their practice.

Hahn [16] describes interrelated modes of socio-cultural creation of events of sickness and healing which include “construction”, “production” and “mediation”. These persistently accompany and complement the pathogenic and therapeutic modes recognised and redressed by biomedicine. Symbols, beliefs, sentiments, rules, thoughts and cognitive schemata as well as rules and standards for interaction are inherent parts of what is described and understood as culture. Hahn [16] goes on to propose the patterned interactions, which guide actions and are judged accordingly by what is defined as the society. These interactions affect conditions of health and suffering. Social definition of illness is the “construction”, whereas socio-cultural influences are mediators, which are perhaps the most recognised and understood socio-cultural influences. According to Hahn [16], the least recognised and understood is the “production”, which is said to be constructed by socially organised interpersonal relationships. It is inevitable that these are strongly influenced by beliefs and relationships which themselves may be pathogenic.

### Disease versus illness

The concept of disease literally means dis-ease, a malfunctioning of a biological or psychic process which is what doctors are trained to deal with, whereas illness is the psychosocial experience as a result of disease, which is what patients are interested in.

## Cultural identity

Apart from their basic cultural identity, individuals carry multiple cultural identities, which are related to a number of factors such as professional background, place of training and place of work. These identities will affect help seeking and world view. These identities are related to the cultural framework we carry within us. Some of these aspects may be easier to give up than others. Acculturation is a complex period of adjustment to the new ideas, attitudes and behaviours through direct or indirect contact with new cultures, which may occur with or without migration. People migrate for a number of reasons within or across countries. Cultural groups themselves are not homogenous groups, and clinicians must ensure that they are aware of these variations. As a result of migration, individuals may experience cultural bereavement as a result of losses [17, 18]). Individuals due to varying cultural values within the same family or across cultures may experience cultural conflict, which has been linked with deliberate self-harm among South Asian females in the UK [19]. Culture shock is the experience that some migrants may face after migration [20] and is defined as an emotional reaction [21].

Training in psychiatry is essentially training residents to become competent clinicians. It is of great importance to take culture into consideration during training to get culturally competent clinicians. However, good clinical practice is about being competent with all the patients irrespective of their cultural upbringing. Cultural competency requires cultural sensitivity, cultural knowledge, cultural empathy, understanding and providing culturally appropriate interactions along with being aware of one’s own cultural strengths and weaknesses. Cultural formulation must include cultural identity of the individual, their beliefs and values, their symptoms in cultural context, their relationship with the environment, factors which reinforce their symptoms, distress as a result of the problems, their explanations for the distress, whether the doctor and the patient have a shared understanding of the problems and a shared plan for addressing the problems, the quality and the nature of the interaction. This is the crux of the training. However, equally importantly the therapists must be aware of their own cultural heritage and whether they are mono-cultural, bicultural or multicultural. They should also be aware of messages they receive from each cultural group and how these messages affect their therapeutic work. Therapists should have their personal abilities to understand and explore their own strengths and weaknesses. They should be acutely  aware of their own world view. And every time they should check  whether it is similar or dissimilar to that of the patient. Trainees must be taught not to be colour blind but be aware of similarities and differences with their patients’ cultural values.

At a “micro-level”, each family system has its own culture. In training to become competent clinicians, it is essential to learn to take the environmental and family context into account.

### Teaching psychiatry in an environmental and family context

Family, as our first socialising agent, for example, serves as a site of both better understanding the origin of mental disturbances and a resource to facilitate personal development. There are many definitions of family including family healthy functioning, dysfunctioning [21] but, according to the scientific epistemology, family is a systemic and network unit. Within particular societies, definitions of family vary depending on cultural and class groupings although as societies become enriched with other cultures their traditional definitions of family are challenged. The evidence-based biopsychosocial formulation of psychiatric disorders and problems is an important element of advanced psychiatric practice. We inevitably deal with families in that practice. We ask about family histories of previous medical and psychiatric diseases, patients’ and family members’ high-risk behaviours (suicide, addiction, eating disorders, etc.), or the use of social media, for example. So we strongly need family intervention and communication skills. There is a difference in the diagnosis and treatment of patients suffering from mental disorders if we treat them individually versus when we treat them as part of larger social (familial) units. The EFCP identifies supporting competencies relevant to family and systemic practice under key competencies that relate to therapy, communication and to the prevention of mental disorder.

It is not an easy task to deal with more than one person at the same time in a room, where the psychiatric examination is taking place. For a psychiatrist in a clinical practice, there is a basic need of interviewing and intervention skills to communicate and help with for not just one but sometimes a group of people around the patient [21]. Family interviewing skills do not mean family psychotherapy. It means a capacity to encourage brief family contact, brief greeting or a 10-min meeting, which develops alliance. It is important for the training of the competent clinician to be familiar with the basic form of family interventions such as psychoeducational family intervention, family consultation, family education, family support, advocacy and self-help group as well as systemic family and network therapy [22].

As well as being adept in working within cultural and family systems, the competent clinician must have undertaken training in psychotherapy as part of their programme [1, 23] so they are able to communicate with the patient and his close environment in a psychotherapeutic fashion.

### Psychotherapy from neglected training to neglected competency for psychiatrists

The UEMS Charter for training in psychiatry [24] stated that experiential training in psychotherapy was a compulsory component of psychiatry training. Of the 45 supporting competencies that underpin the five key competencies that compose the EFCP metacompetency of the physician as psychiatric expert, seven explicitly refer to psychotherapeutic content. In addition, six of the twelve supporting competencies that underpin the metacompetency of the physician as communicator explicitly or implicitly draw upon knowledge and skill that are acquired through the use of psychotherapeutic methods.

Psychiatrists start their basic training within a largely biologically oriented, general medical framework, bringing with it the risk of “professional alexithymia” [24]. The (relatively) successful clinical use of psychopharmaceutical agents contributed to a decline in psychotherapy training and practicing for psychiatrists. Miller [25] noted the “apparent atrophy of psychotherapy skills among recent graduates” (p. 129). This suggests that an important element of psychiatry training is not being fully delivered.

Trainees in psychiatry for their part consider that psychotherapy should be an integral part of their professional identity, in the USA [26], as well as in Europe [27]. The training requirements of this are not easy to implement [28].

In some countries (e.g. the Flemish part of Belgium), psychiatrists devote between 30 and 100 % of their time to psychotherapy [29], which may resemble the situation in most of the southern European countries. This skewed distribution of psychotherapy in psychiatric practice illustrates another issue namely the differences in emphasis in psychiatry on the major psychotherapeutic schools at present. Where in most countries, the focus lays on evidence-based forms of psychotherapy: behavioural (cognitive) and family therapy, in the countries where psychiatrists devote more time to psychotherapy, a third school namely psychoanalysis favoured.

Although in many western and northern European countries, psychotherapeutic skills are considered important as one of the competences to enable psychiatrists to communicate well with their patients and support them adequately, formal psychotherapy is left in the hands of well-trained psychotherapists, mostly clinical psychologists. The harmonisation of psychotherapy training is therefore a great challenge. Initiatives, such as teaching empathy to medical undergraduates might be an important first step [30]. Standardising existing training programmes [31] may be a second step.

The discussions should no longer revolve around the effectiveness of specific psychotherapies, or whether residents in psychiatry should have formal psychotherapy training, but rather about how psychotherapy should be implemented in psychiatric practice *together with other therapies* such as pharmacotherapy, ECT, deep brain stimulation, light therapy. The focus should be on the challenge of teaching trainees about these approaches in an integrated manner [32, 33].

Once we have arrived at consensus regarding the content of what is needed to encourage the training of the competent clinician, the next challenge is to develop processes to assure the public and others, that there is evidence that the standards of our training are being harmonised. This can be examined in two ways: looking for evidence of the impact of initiatives to harmonise training and looking at the assessment of the outcomes of training. There are major challenges in both of these areas.

### The impact of initiatives to harmonise training

The harmonisation of various aspects of psychiatric training is problematic. Achieving this entails the incorporation of European recommendations within national programmes and implementation of these programmes by local educational providers.

At present, there is only a small literature on the impact of initiatives to harmonise the training of psychiatrists in Europe [9, 34]. These are almost all descriptive studies and opinion pieces. However, the results of these studies are consistent. In general, the studies find that the most problematic areas are in psychotherapy training, lack of mentorship for trainees and in the variability of approaches to quality assurance of training and in the assessment of outcomes of training.

### Why has the harmonisation project failed to deliver?

As Mayer et al. [1] concluded and our viewpoint concurs, the project to harmonise psychiatry training in Europe has failed to harmonise training, despite the will and effort of a good number of the great and the good of the European psychiatric establishment. A framework derived from the arena of organisational behaviour may help us to understand the failure and to engage in work to remedy the situation.

William Ouchi [35] conducted a classic review of theories and evidence that explain what he called “the problem of cooperation”, that is how we gain a sociological understanding of the factors that influence human beings to align their behaviours behind shared goals. The issue of harmonisation of training standards appears to us to be a case that exemplifies “the problem of cooperation”, that is how to align systems of psychiatry training around shared standards. From his analysis, Ouchi was able to distinguish and describe three mechanisms in which organisational systems sought to influence the behaviours of people and other organisations: he termed these mechanisms markets and bureaucracies and clans. He went on to theorise about the conditions in which each of the mechanisms would be most effective. He acknowledged that in the real world, none of the mechanisms existed in pure form; reality throws up combinations of the three; nevertheless, his descriptions can be applied in many situations.

According to Ouchi, a market form of influence is characterised by an economic transaction, such as when a person exchanges a unit of labour for money. The optimum conditions for this are when there is a balance of power between the parties to the exchange and the exchange can have a price assigned to it. A bureaucracy operates when there is a legitimate and enforceable line of authority between the parties, and there are explicit rules to be followed. Clan forms of influence are characterised by systems in which there are shared values and beliefs and traditions of practice control behaviours.

From this description, it can be seen that the harmonisation project has sought to use the tools of bureaucratic influence, that is, through setting rules such as standards and frameworks. Critically, there has been an absence of legitimate enforceable authority between the bodies setting the rules and those that are meant to follow them. In short, the regulatory body has no power. This arrangement may be termed a pseudo-bureaucracy.

Unfortunately, many of the recommendations that have been made to address the problems with the harmonisation project as put forward by Mayer et al. [1] may be categorised as pseudo-bureaucratic and are therefore likely to fail. For example, the suggestion of having a European Board qualification in psychiatry is superficially attractive. When looking at the majority of countries in Europe where assessment methods are comparable, assessment structures for psychiatric trainees differ substantially. For example, in the UK or the Netherlands, a structured and comprehensive competency-based assessment is applied, while in some countries, the final assessment consists of a trainer evaluation only. Introducing a European qualification is a typical bureaucratic response to the problem, involving standardisation and evidencing. There is, however, no regulatory compulsion for trainee psychiatrists to hold this qualification, so it lacks the legitimate authority that is an essential element of a bureaucratic control. Furthermore, it would fail to satisfy the criteria necessary for it to constitute an element of a market control. The under-supply of psychiatrists in Europe is not going to stimulate a market drive for doctors to hold the qualification. There is simply no need for the few psychiatrists in Europe to increase their market value by enhancing their qualifications.

Clan control may offer some hope for the project. Medicine is an ancient profession with a strong tradition of teaching the next generation. From the ancient form of the Hippocratic oath to its modern form as reformulated by Lasagna [36] in 1964, “and gladly share such knowledge as is mine with those who are to follow”, medical professionals have had an obligation to teach and to train. The issue for us is how to harness this tradition in the service of harmonising psychiatry training. The key to enhance clan control is to encourage the socialisation of individuals into the values, norms and expectations of the organisation [35] in short to put in place initiatives that develop a culture that encourages the delivery of uniformly high standards of training.

The participation of a wider range of stakeholders in the conversations that shape psychiatry training can help the process of culture change that may influence improvements in learning in psychiatry in Europe. Two such groups are psychiatrists in training as recently qualified specialists.

### A view from within: the involvement of psychiatrists in training

The involvement of psychiatrists in training in the development of national programmes and in the quality assurance of training varies significantly around Europe [38]. The European Federation of Psychiatric Trainees (EFPT) represents the consensus of psychiatric trainees associations across more than 30 European countries, advocating for the improvement in psychiatry training [37] This federation has been offering trainees the opportunities to have an exchange in other European country, experiencing a different training programme and mental health system, having as a priority to have direct feedback from psychiatric trainees, analysing the concerns related to psychiatry and training across Europe [38].

Surveys of psychiatrists in training conducted by the EFPT show that the main concerns around Europe revolve around discrepancies between the stated national programme and the lived experience of trainees especially around delivery of specific training opportunities in aspects of service delivery, in research methodologies and in psychotherapy. Levels of recruitment into psychiatry and inadequate working conditions also concern trainees [28, 39]. At a time of economic challenge, trainees are worried about wider resourcing issues for mental health, including the funding of training, duration of working hours and pressure on the whole mental health workforce, creating a dysfunctional environment affecting the quality of training [40].

Another group who may be involved as agents for change in the endeavours to harmonise and to improve psychiatry training is those who have recently completed training, the early career psychiatrists.

### The early career psychiatrist

Despite the changes that have taken place in psychiatric practice in recent decades, much specialist training and continuous medical education in Europe continue to be based on old-fashioned paradigms [9] and do not fully equip the newly qualified specialist for contemporary practice as a competent clinician. Several actions have been taken by national and international bodies to describe the gap between training and practice of early career psychiatrists. In particular, the Early Career Psychiatrists’ Committees of the European Psychiatric Association (EPA) and of the World Psychiatric Association (WPA) have carried out several surveys in different European countries to identify the areas with the most significant educational needs and have suggested actions to fill such gaps. The areas identified include psychopathology, psychotherapy, prevention and early intervention, drug management and treatment of physical diseases in patients with mental disorders [41, 42].

Early career psychiatrists have reported other shortcomings about their training, including the lack of practical knowledge to manage the transition phase from residency to practice [42] managing the risk of burn out, and skills for dealing with the media, being involved with professional and scientific societies and for handling difficult patients and colleagues.

European early career psychiatrists have recently proposed a working agenda [42] with the aim of defining a new identity for the modern psychiatrist and to produce a curriculum for early career psychiatrists. The topics in the proposed agenda seek to address the shortcomings in training that they have experienced and the issues that they have encountered as they made the transition to specialist.

## Conclusions and recommendations

Psychiatry is the largest medical specialty in Europe, but by no means yet are there common standards nor requirements for training and certification that are recognised and implemented in all countries across Europe. The reasons are diverse, but cultural and political insights and influences account for these differences.

Standardisation of training and certification requirements is important to ensure quality and patient safety and facilitate the free movement of psychiatrists across the European community. However, as our analysis of cultural factors begins to explore, it is important that the drive for harmonisation produces psychiatrists who are capable of transferring their skills into different social and cultural situations and it avoids an approach that sacrifices diversity for the sake of a misplaced standardisation. As in other specialties, a standardised European board examination could be considered as a goal to pursue, which may at least give some assurance by testing the presence of the core knowledge that is essential for the practice of psychiatry in all contexts.

On the short term, UEMS should join forces not only with the National Psychiatric Associations united in the Council of NPA’s within the European Psychiatric Association, but also with the users the trainees and early career psychiatrist (EFPT) and consumers organisations to discuss and establish a road map for implementing a realistic and responsible standard for training and (re)certification of psychiatrists across Europe.

## Footnotes

1 The seven physician roles are Psychiatric Expert/Clinical Decision-Maker, Communicator, Collaborator, Manager, Health Advocate, Scholar and Professional.
